# Supplementary material for: An extracellular vesicle delivery platform based on the PTTG1IP protein
Source: Extracell Vesicle. 2024 Dec;4:None. doi: 10.1016/j.vesic.2024.100054 (PMC11655445; doi:10.1016/j.vesic.2024.100054)
Supplement: Multimedia component 1 [file mmc1.docx]

**Supplementary Information**

| Application | Antibody | Host | ID | Manufacturer | Dilution |
| --- | --- | --- | --- | --- | --- |
| Primary | Anti-GFP | Rabbit | ab290/ 338001 | Abcam/Biolegend | 1:1,000 |
| Primary | Anti-cMyc | Mouse | 626801 | Biolegend | 1:1,000 |
| Primary | Anti-CD63 | Mouse | Ab1289 | Abcam | 1:1,000 |
| Primary | Anti-ACTB | Mouse | MA5-15739 | ThermoFisher | 1:2,000 |
| Primary | Anti-ALIX | Rabbit | ab186429 | Abcam | 1:2,000 |
| Secondary | Anti-Rabbit | Goat | 926-32211 | LI-COR | 1:5,000 |
| Secondary | Anti-Mouse | Goat | 926-32210 | LI-COR | 1:5,000 |

**Table S1**

**Details of antibodies used in this study.**


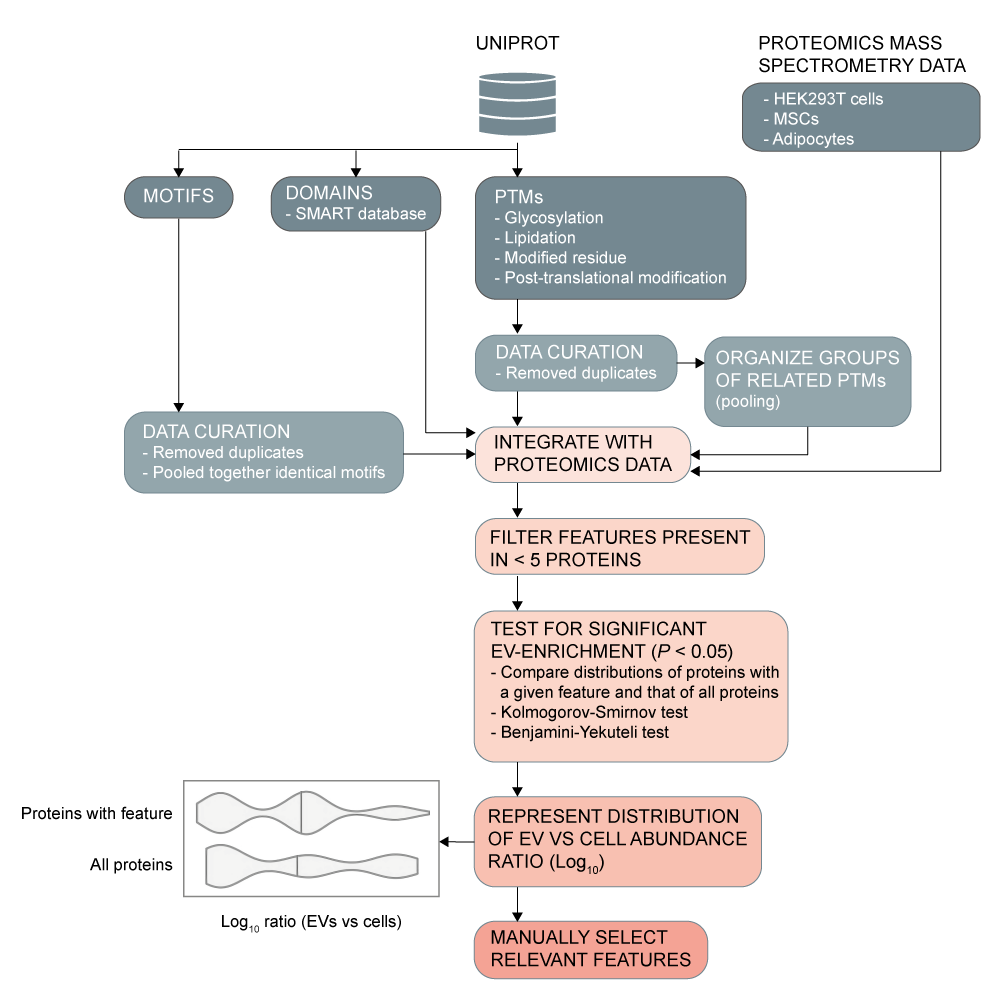


**Figure S1**

**Bioinformatics analysis scheme.**

Protein motif, domain (as annotated on SMART database), and post-translational modification (PTM) data were extracted from the UniProt database. Motif and PTM data were then manually curated to remove duplicates and pool together identical features. In the case of PTMs, additional groups of related PTMs were pooled and included in the analysis as separate categories. Protein feature data were integrated with mass spectrometry proteomics data325 (from HEK293T cells, MSCs, and adipocytes) and only features that appear on at least 5 proteins were included in the analysis because of the difficulty to detect statistically significant changes of feature annotations when data was scant. Distributions of proteins with a given feature and that of all proteins were then compared by Kolmogorov-Smirnov test followed by Benjamini-Yekuteli post hoc test to identify features that were significantly EV-enriched (P < 0.05). The distribution of EV vs cell abundance ratio (log_10_) of proteins with significantly EV-enriched features was then represented and relevant features were manually selected.


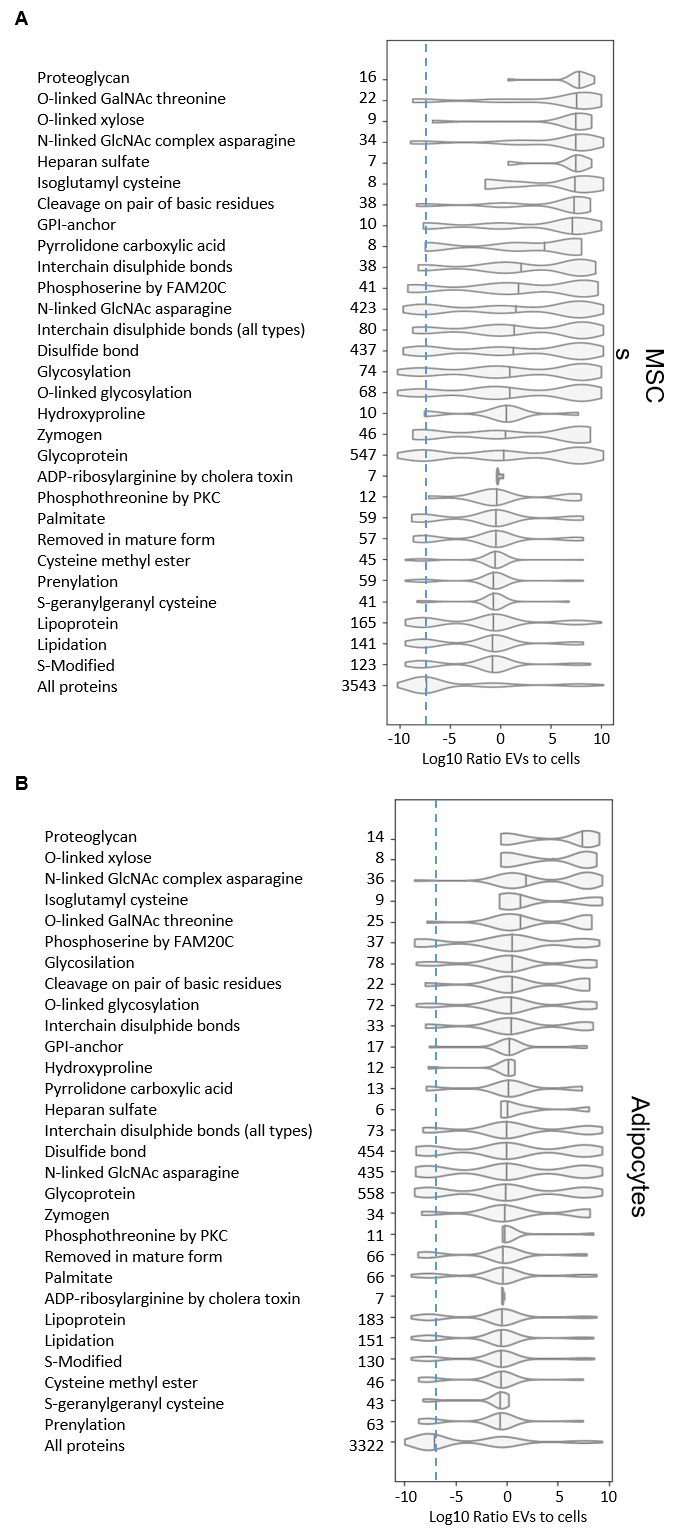


**Figure S2**

***In silico*-identified EV-enriched PTM annotations in MSCs and adipocytes.**

Violin plots representing the EV to cell abundance ratios (log_10_) of proteins with statistically significant EV-enriched PTMs identified in (**A**) MSCs and (**B**) adipocytes. Only EV-enriched annotated PTMs common across all three cell lines are shown. Numbers on the y-axes depict the number of proteins with the given PTM in the dataset. Violin plot of the EV to cell abundance ratios (log_10_) of all proteins in the dataset is included as a reference. Median value of all proteins in the dataset is represented as a dashed line. **P* < 0.05.


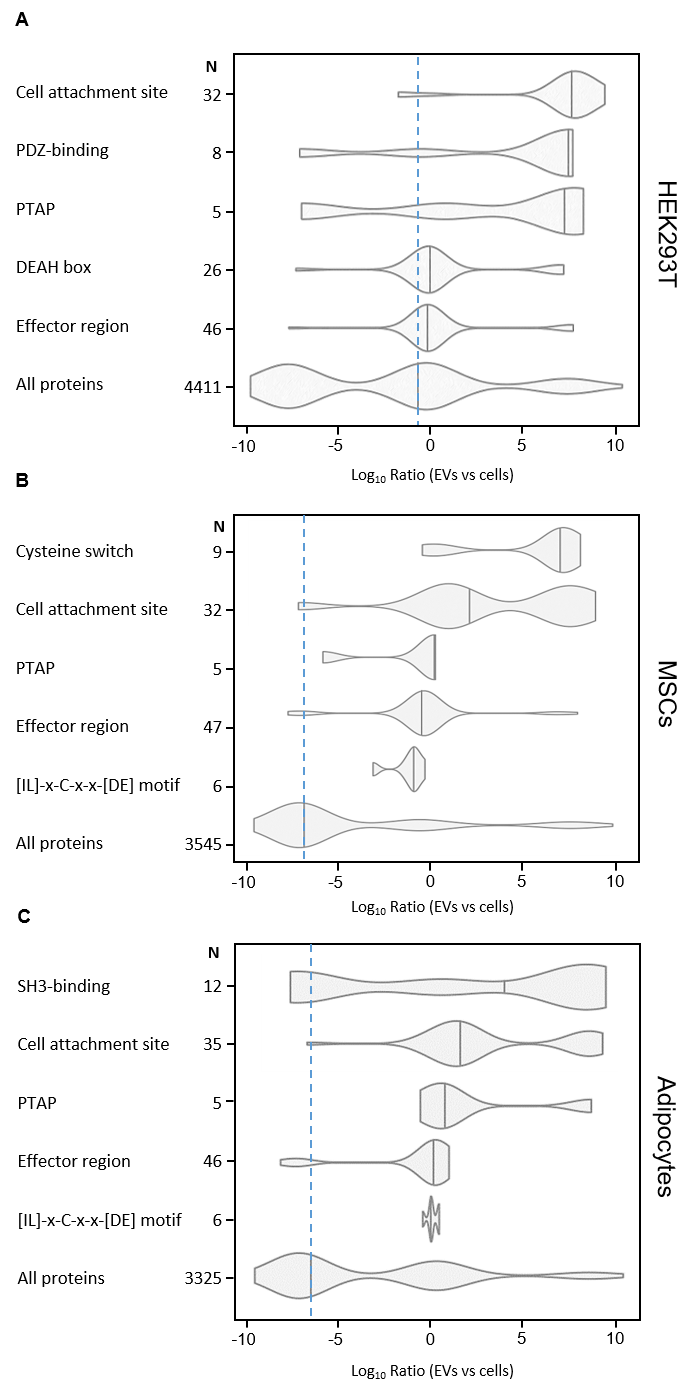


**Figure S3**

***In silico*-identified EV-enriched annotated motifs.**

Violin plots representing the EV to cell abundance ratios (log_10_) of proteins with statistically significant EV-enriched annotated motifs identified in HEK293T cells (**A**), MSCs (**B**) and adipocytes (**C**). Numbers on the y-axes depict the number of proteins with the given motif in the dataset. Violin plot of the EV to cell abundance ratios (log_10_) of all proteins in the dataset is included as a reference. Median value of all proteins in the dataset is represented as a dashed line. **P* < 0.05.


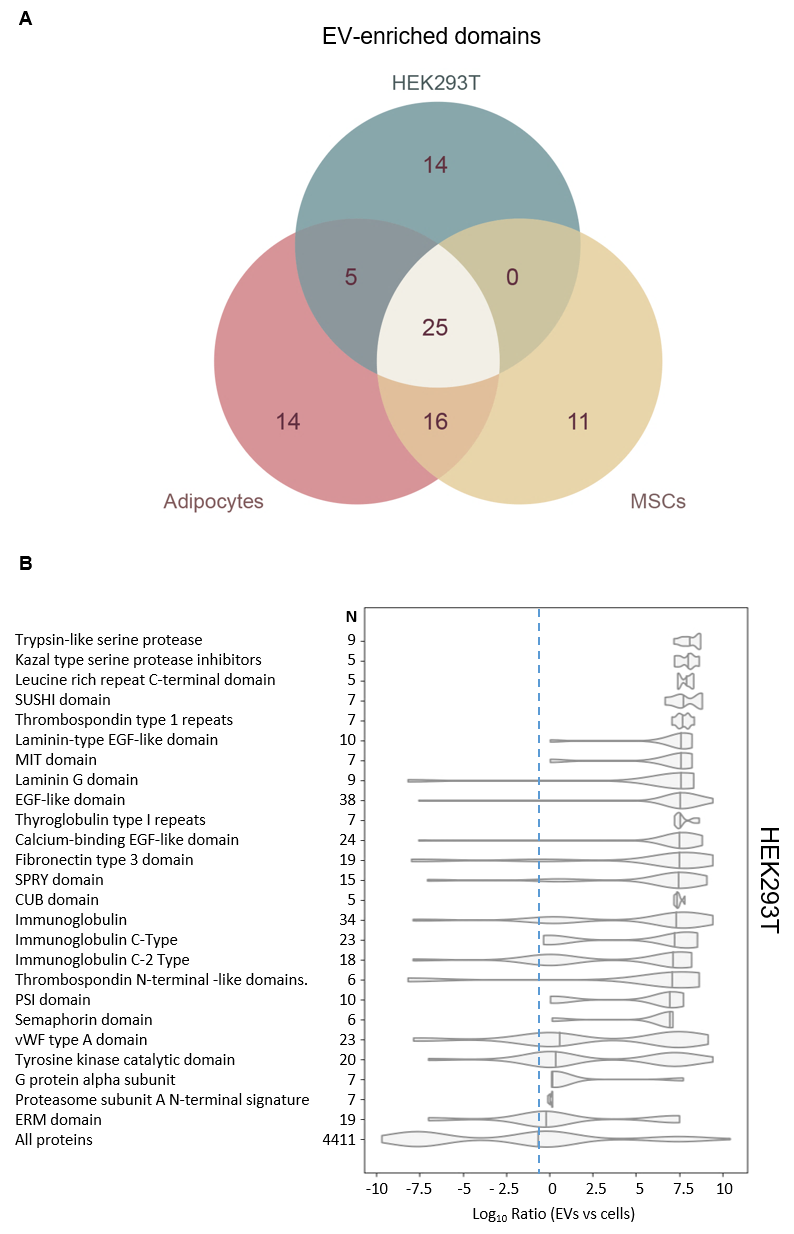

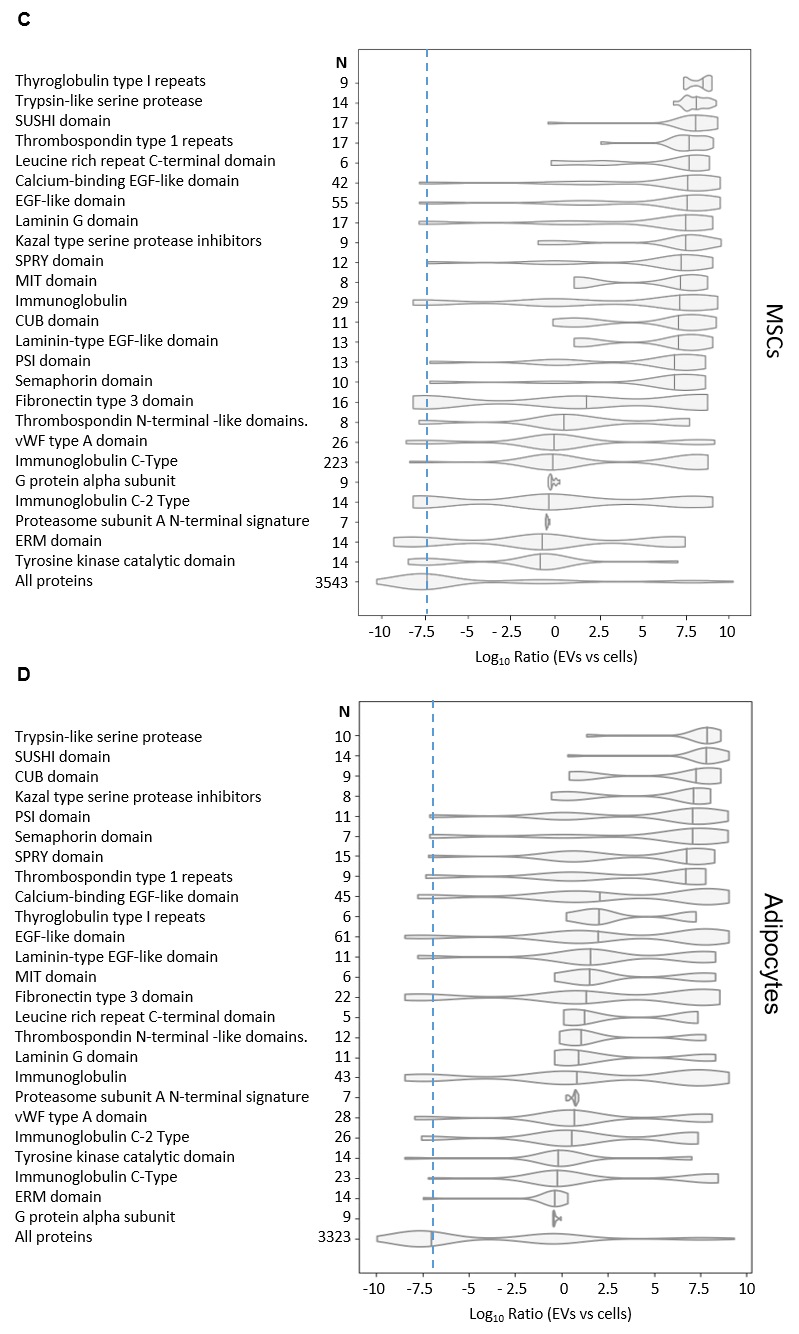


**Figure S4**

***In silico*-identified EV-enriched domain annotations.**

(**A**) Venn diagram of statistically significant EV-enriched annotated domains in HEK293T cells, MSCs and adipocytes. Numbers in the diagram refer to the number of domains identified for each group. Violin plots representing the EV to cell abundance ratios (log_10_) of proteins with statistically significant EV-enriched domains identified in HEK293T cells (**B**), MSCs (**C**) and adipocytes (**D**). Only EV-enriched annotated domains common across all three cell lines are shown. Numbers on the y-axes depict the number of proteins with the given domain in the dataset. Violin plot of the EV to cell abundance ratios (log_10_) of all proteins in the dataset is included as a reference. Median value of all proteins in the dataset is represented as a dashed line. **P* < 0.05.


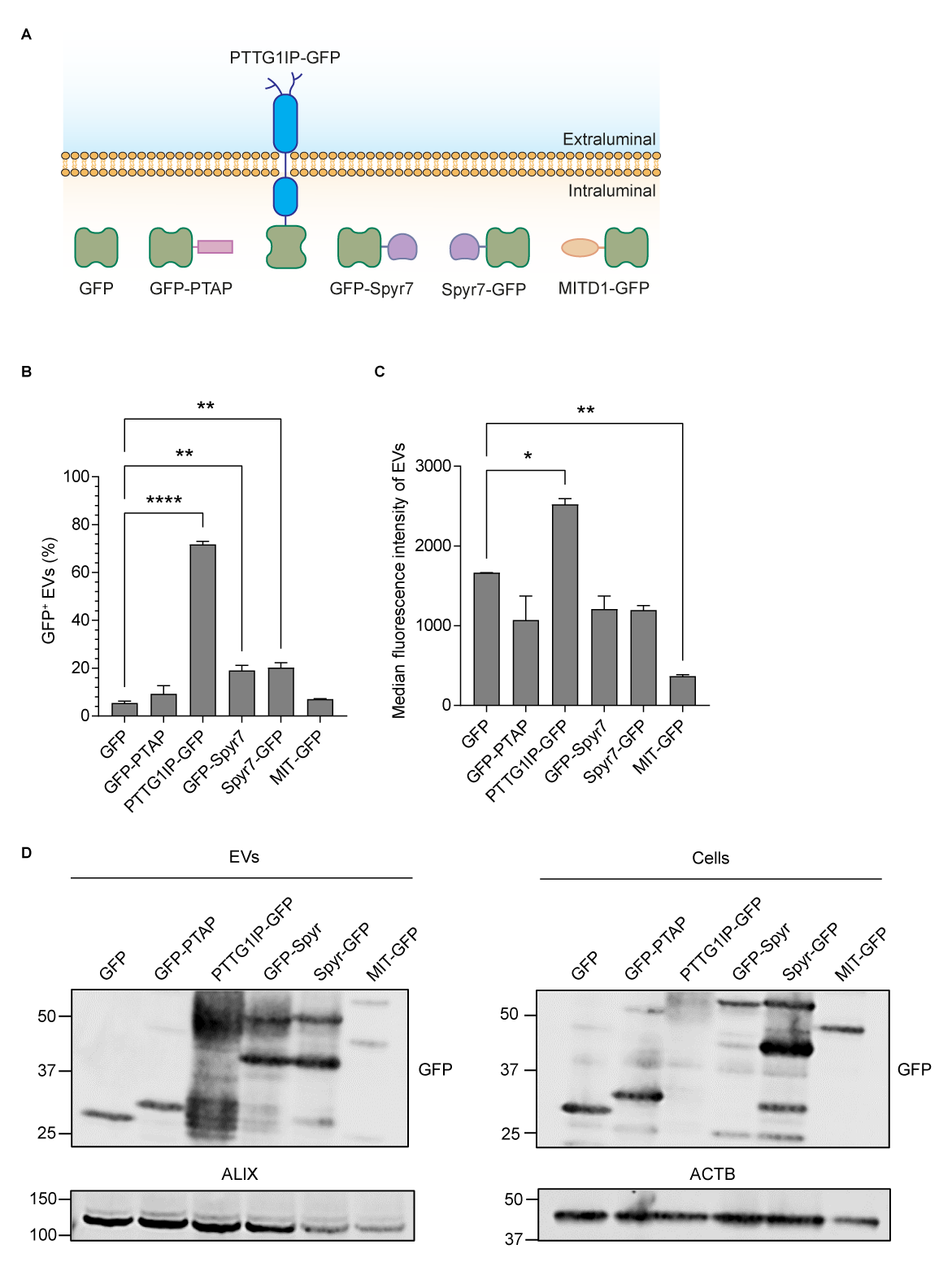


**Figure S5**

**Assessment of EV-loading features from bioinformatics analysis.**

(**A**) Scheme of the GFP-fusion constructs that were assessed. (**B**) Percentage of GFP^+^ EVs quantified by single-vesicle analysis of conditioned media from HEK293T cells transfected with GFP-fusion constructs. (**C**) Median fluorescence intensity of GFP^+^ EVs. (**D**) Western blot of GFP-fusion constructs in HEK293T cells and EVs. Cell lysates and isolated EVs were blotted against ACTB and ALIX respectively. Both cell lysates and isolated EVs were blotted against GFP. Statistical significance was tested by one-way ANOVA and Bonferroni post hoc test. Data are presented as mean + SEM, n = 3 replicates, *P < 0.05, **P < 0.01, ****P < 0.0001.


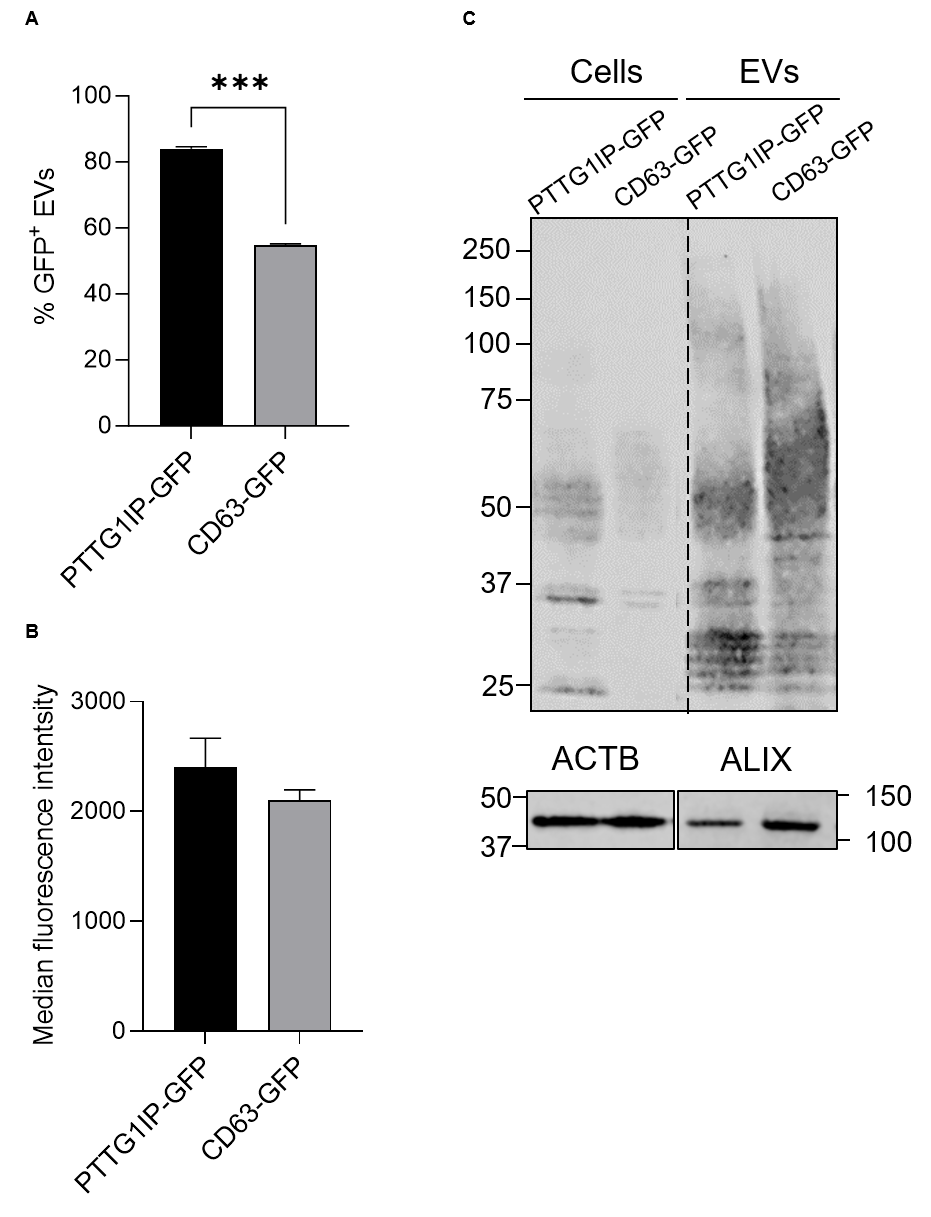


**Figure S6**

**PTTG1IP has a higher EV-loading capacity than CD63.**

PTTGIP and CD63 EV-loading was compared by single vesicle analysis and Western Blot. (**A**) Percentage of GFP^+^ EVs quantified by single-vesicle analysis of conditioned media from HEK293T cells transfected with PTTG1IP-GFP or CD63-GFP. (**B**) Median fluorescence intensity of GFP^+^ EVs quantified by single-vesicle analysis. (**C**) Western blot of GFP-fusion constructs in HEK293T donor cells and EVs. Cell lysates and isolated EVs were blotted against ACTB and ALIX respectively. Both cell lysates and isolated EVs were blotted against GFP. Statistical significance was tested by Student’s *t*-test. Data are presented as mean + SEM, *n* = 3 replicates, ****P* < 0.001.


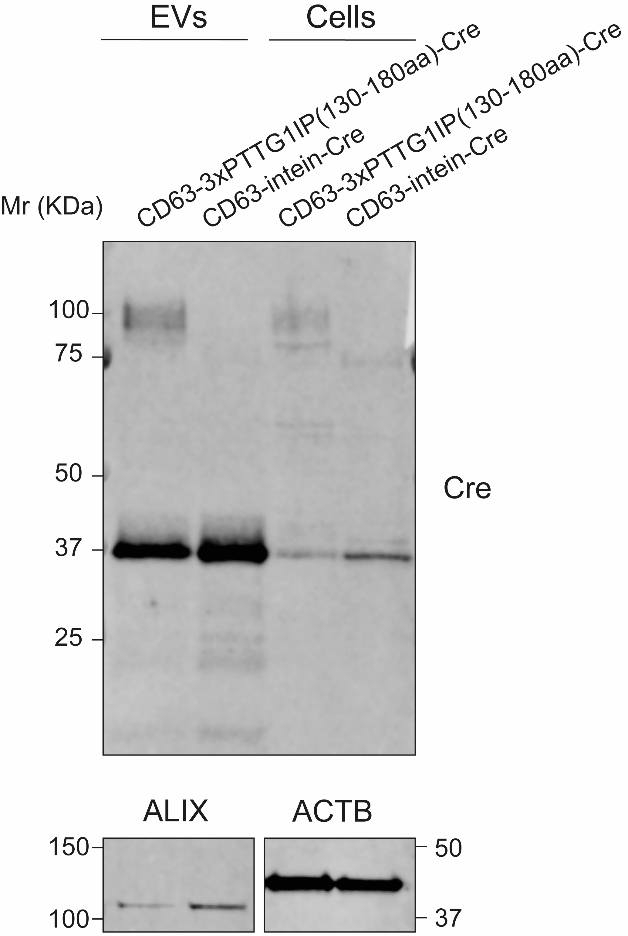


**Figure S7**

**Amino acids 130-180 of PTTG1IP are able to induce cleavage in EVs when fused to CD63.**

HEK293T cells were transfected with CD63-intein-Cre or CD63-3xPTTG1IP(130-180aa)-Cre, and Cre cleavage in the EVs was analyzed. Western blot of CD63-intein-Cre and CD63-3xPTTG1IP(130-180aa)-Cre in HEK293T cells and EVs. Cell lysates and isolated EVs were blotted against Cre, ALIX and ACTB.

**Figure S8**

**Assessment of Cas9 reporter cell activation after Cas9 and sgRNA transfection.**

GFP positive cells quantified by flow cytometry 48h after Cas9, sgRNA or Cas9 and sgRNA transfection to HEK293T stoplight reporter cells. Statistical significance was tested by one-way ANOVA and Bonferroni post hoc test. Data are presented as mean + SEM, n = 3 replicates, ****P < 0.0001.

**Figure S9**

**Comparison of PTTG1IP and CD63 for EV-mediated delivery of Cas9.**

GFP positive cells quantified by flow cytometry 48h after PTTG1IP-intein-Cas9 or CD63-intein-Cas9 EV-transfer to sgRNA transfected HEK293T stoplight reporter cells. Isolated EVs were transferred to recipient cells in 96-well plates (2×10^9^ EVs/ml). Statistical significance was tested by Student’s *t*-test. Data are presented as mean + SEM, *n* = 2, ***P* < 0.01.


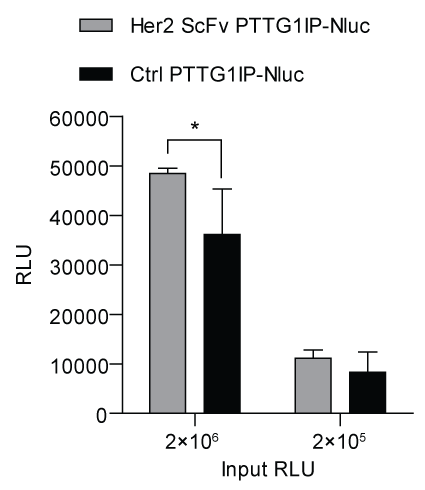


**Figure S10**

**Uptake of targeted EVs displaying HER2 scFv in PTTG1IP N terminus by human breast cancer cell line SKBR3**.

Luciferase activity (RLU, relative light units) in recipient SKBR3 cells after treatment with EVs (2×10^6^ or 2×10^5^ input RLUs) derived from HEK293T cells expressing PTTG1IP fused to HER2 scFv targeting domain at the N terminus and to Nluc at the C terminus, or EVs derived from cells expressing PTTG1IP fused to only to Nluc. Values are mean + SEM, *n* = 3. **P* < 0.05 (one-tailed Student’s *t*-test).
